# Characterization of the Fecal Microbiome in Dogs Receiving Medical Management for Congenital Portosystemic Shunts

**DOI:** 10.3389/fvets.2022.897760

**Published:** 2022-07-28

**Authors:** Nathan Squire, Cassie Lux, Katie Tolbert, Jonathan Lidbury, Xiaocun Sun, Jan S. Suchodolski

**Affiliations:** ^1^Department of Small Animal Clinical Sciences, University of Tennessee College of Veterinary Medicine, Knoxville, TN, United States; ^2^Department of Veterinary Small Animal Clinical Sciences, Texas A&M College of Veterinary Medicine and Biomedical Sciences, College Station, TX, United States; ^3^Office of Information and Technology, University of Tennessee Knoxville, Knoxville, TN, United States

**Keywords:** dysbiosis index, hepatic encephalopathy (HE), lactulose, quantitative polymerase chain reaction (qPCR), portosystemic shunt (CPSS)

## Abstract

**Background:**

The GI microbiome has not been characterized in dogs being medically managed for congenital portosystemic shunts (CPSS).

**Objectives:**

To characterize the fecal microbiome in a population of dogs being medically managed for CPSS.

**Animals:**

27 client-owned dogs.

**Methods:**

Prospective cohort study enrollment of fecal samples was performed with follow-up data collected retrospectively. The overall fecal dysbiosis index (DI) and individual bacterial abundances were determined using real-time qPCR. Medical management, clinical findings, clinicopathologic, and outcome variables were collected, and logistic regression analyses were performed to evaluate associations between these variables and overall DI and bacterial abundances. Numerical variables were evaluated with general linear models for normality and equal variance using Shapiro-Wilk test and Levene's test, respectively.

**Results:**

All dogs were administered a hepatic diet and lactulose, while antibiotics were used in 22 (81.5%) and acid suppressants in 7 (25.9%). Seventeen dogs (63.0%) had a DI >2. The median DI in this population was 3.02 (range 4.23–8.42), and the median DI in dogs receiving and not receiving antibiotics was 4.3 (range −4.23–8.42) and 1.52 (range −1.62–5.43), respectively. No significant association between any of the analyzed variables and the DI was identified. There was a significant association between the use of metronidazole and a larger abundance of E. coli (*p* = 0.024).

**Conclusions and Clinical Importance:**

Dysbiosis appears to be common in dogs that are being medically managed for CPSS, though the clinical significance remains unclear.

## Introduction

The microbiota of the mammalian gastrointestinal (GI) tract consists of trillions of organisms performing a variety of crucial roles both locally and systemically (1–3). Characterization of the GI microbiome has evolved from culture-based models to high-throughput DNA sequencing techniques or targeted quantitative polymerase chain reaction (qPCR) assays, which have allowed for the characterization of the microbiome at the genetic and species level in various species (3–5).

Several studies have characterized bacterial populations for the GI flora in healthy humans and animals (1–3). Subsequently, extensive research has been performed evaluating the GI microbiome characteristics in human and canine populations with specific pathologies such as chronic inflammatory enteropathies, diabetes, obesity, and hepatopathy-induced hepatic encephalopathy (HE) (6–10). By using the previously identified healthy control profiles of intestinal microbiota in comparison to the profiles from the diseased populations, the concept of a “dysbiosis index” (DI) was established, which focuses the analysis of shifts in the microbiome to certain important bacterial groups.4 Certain alterations in the GI microbiome have been postulated to be a useful adjunct for identifying disease states or a potential monitoring parameter for treatment efficacy (4–8, 11).

The role of the GI microbiome in chronic hepatopathies and HE has been frequently investigated in humans, with ammonia being identified as a key player (10, 12, 13). Significant differences have been reported in humans between the colonic microbiota of cirrhosis or HE patients and healthy control individuals, and some suggest the microbiome alterations in affected patients may contribute to more significant clinical signs of HE *via* alteration of the intestinal barrier function, an increased proportion of urease-producing bacteria, or in other ways (11–14). Therefore, it has been postulated that manipulation of the microbiome may contribute to improved outcomes in these patients.

In dogs, the most common cause of HE is related to congenital portosystemic shunts (CPSS) (15). Single or multiple aberrant blood vessels allow portal venous blood to bypass the liver parenchyma with CPSS (15, 16). Medical management for dogs with PSS can include a restricted protein diet and administration of lactulose, antibiotics, anticonvulsants, or probiotics. Surgical attenuation of the CPSS is often recommended to restore more normal portal blood flow and improve liver function. Some individual components of medical management for CPSS have been investigated for effects on the microbiome in dogs (4, 15, 17–22). Based on the literature, it is apparent that lactulose, diet, and certain antibiotics have the potential to alter the microbiome; however, these studies investigate effects in healthy dogs, people, or non-hepatic disease (11, 15–20). Because dogs being medically managed for CPSS are often prescribed these medications for long periods, this may lead to long term dysbiosis and potential subsequent health consequences.

There is a deficit in the literature as to the effects of these medications on the fecal microbiome and DI in dogs with CPSS and a complete lack of information as to whether these effects have any consequences on the clinical signs or outcomes of dogs with CPSS.

The aims of the current study were to characterize dysbiosis in dogs being medically managed for CPSS and to evaluate for associations between dysbiosis and clinical variables such as medical management, clinical signs, clinicopathologic findings, and postoperative outcomes. We hypothesized that medical management for CPSS would have a significant impact on the dysbiosis index, but that clinical outcome of the patient would not be affected regardless of the level of dysbiosis.

## Materials and Methods

### Case Selection

Dogs presenting to the University of Tennessee College of Veterinary Medicine (UTCVM) with a previous diagnosis of CPSS (confirmed either with transsplenic portal scintigraphy or computed tomography) were prospectively recruited from June, 2018 to August, 2019.

### Inclusion Criteria

Dogs were included in the study following definitive diagnosis of CPSS using either CT/angiography or transsplenic nuclear portal scintigraphy, and if fecal samples were able to be collected at the initial visit prior to surgical intervention for CPSS. All animals were enrolled following informed owner consent in accordance with the protocol approved by the University of Tennessee Institutional Animal Care and Use Committee (AUP #2641-0918). Animals were excluded if the fecal sample was not sufficient to be analyzed, if a fecal sample was not obtained, or if a fecal sample was not obtained prior to administration of perioperative antibiotics for the CPSS attenuation procedure.

### Medical Record Review

Information regarding patient signalment (breed size, sex, age, and reproductive status), weight, clinical signs prior to medical management (GI signs, neurologic signs, urinary signs), medications at the time of sample collection, clinical signs following initiation of medical management, preoperative clinicopathologic findings (complete blood count (CBC), serum biochemistry panel, pre- and post-prandial serum bile acids, and resting plasma ammonia), type of surgical intervention, postoperative clinical outcome, and postoperative albumin, BUN, cholesterol, and glucose values. Postoperative clinicopathologic and outcome data was obtained *via* a combination of medical record review and communication with referring veterinarians. Clinical management of the dogs was not altered for the study parameters, and each dog was managed at the discretion of the attending clinician, including adjustments to medical management perioperatively or postoperatively and surgical attenuation. In general, each dog was recommended to have a follow-up serum biochemistry panel, CBC, and pre- and post-prandial serum bile acids performed between 3 and 6 months postoperatively. Neurologic signs included seizures, ataxia, head pressing, or mental obtundation. Gastrointestinal signs included vomiting, diarrhea, or regurgitation, and were considered distinct from anorexia or hyporexia. Lower urinary tract signs included hematuria, stranguria, or dysuria.

Closure of the CPSS following surgical intervention was suspected based on the following findings in follow-up blood analyses: normalization or improvement of serum bile acids22 with improvement or normalization of hepatic synthetic factors such as albumin, glucose, cholesterol, or BUN.

### Sample Collection

Fecal samples were either collected *via* free-catch fecal samples, digital rectal exam, or a fecal loop, and stored in a cryogenic storage container. Following fecal collection, the samples were immediately stored in a −80°C freezer until all samples for study inclusion were collected and shipped for analysis. Samples were then shipped on dry ice and maintained in a −80°C freezer at the Texas A&M Veterinary Medical Diagnostic Laboratory until the molecular diagnostics were performed.

### Fecal Quantitative PCR Analysis

DNA was extracted from each fecal sample (100 mg) using the MoBio Power soil DNA isolation kit (MoBio Laboratories, USA) according to the manufacturer's instructions. The qPCR assays were performed as previously reported by AlShawaqfeh et al. In summary, qPCR reactions were performed using SYBR green-based reaction mixtures. The final total reaction volume was 10 μL. The final mix was composed of 5 μL SsoFast EvaGreen supermix (Bio-Rad Laboratories, CA, USA), 0.4 μL each of a forward and reverse primer, 2.6 μL of PCR water and 2 μL of normalized DNA. The PCR conditions were as follows: initial denaturation at 98°C for 3s and annealing for 3s. Melt curve analysis was performed post-amplification using these conditions: 95°C for 1 min, 55°C for 1 min and increasing incremental steps of 0.5°C for 80 cycles for 5s each. All samples were run in duplicate fashion. The qPCR data were expressed as the log amount of DNA (fg) for each particular bacterial group/10 ng of isolated total DNA. Each fecal sample was evaluated for the overall bacterial number, as well as the individual bacterial species including: Universal Bacteria, Faecalibacterium, Turicibacter, Streptococcus, E. coli, Blautia, Fusobacterium, and C. hiranonis. The overall dysbiosis index was then calculated for each dog as previously described by AlShawaqfeh et al. (4).

The reference ranges for the bacteria tested to determine if the abundance was normal or abnormal were expressed as log DNA/gram of feces, and included Universal Bacteria (10.6–11.4), Faecalibacterium (3.4–8.0), Turicibacter (4.6–8.1), Streptococcus (1.9–8.0), E. coli (0.9–8.0), Blautia (9.5–11.0), Fusobacterium (7.0–10.3), and C. hiranonis (5.1–7.1); these reference ranges were adapted from previous work by AlShawaqfeh et al. (4). The DI was classified as normal DI (<0), a moderate shift (0–2), or a significant shift (> 2).

### Statistical Analysis

Normality tests were conducted on numeric variables using Shapiro-Wilke tests. Descriptive statistics were calculated. Normally distributed data are presented as a mean ± SD, and non-normally distributed data are expressed as median and range. Logistic regression analysis was used to evaluate the effects of clinical variables (clinical signs, medical management, clinicopathologic values, and outcome data) on the categorical outcomes of bacterial abundance (normal or abnormal) and DI (normal, equivocal, or dysbiosis). The effects of clinical variables on numeric outcomes (such as clinicopathologic data) were evaluated using general linear models. Diagnostic analysis was conducted to examine model assumptions for normality and equal variance using Shapiro-Wilk test and Levene's test respectively. Ranked transformation was applied if diagnostic analysis exhibited violation of normality and equal variance assumptions. *Post hoc* multiple comparisons were performed with Tukey's adjustment. Statistical significance was identified at the level of 0.05. Analyses were conducted in SAS 9.4 TS1M7 for Windows 64x (SAS institute Inc., Cary, NC).

## Results

A total of 27 dogs were included in the study in accordance with the inclusion criteria. The study population included 17 (63.0%) male dogs and 10 (37.0%) female dogs. Ten (58.8%) male dogs were neutered, and 6 (60%) female dogs were spayed. Median age at the time of fecal collection was 10 months (range, 3–48). Median body weight at the time of collection was 4.9 kg (range, 1.5–32.8).

Prior to initiation of medical management, documented clinical signs in the dogs of the study included neurologic signs in 16 (59.3%), gastrointestinal signs in 13 (48.1%), episodic or persistent anorexia or hyporexia in 11 (40.7%), and lower urinary tract signs in five (18.5%).

At the time of fecal collection, medical management had been instituted for all dogs. Medical management consisted of varying combinations of a therapeutic hepatic diet (all dogs), lactulose (all dogs), antibiotics in 22 (81.5%), acid suppressants in 7 (25.9%), antiepileptics in three (11.1%), and probiotics in two (7.4%). The antibiotics most commonly prescribed were metronidazole in 17 dogs receiving antibiotics (77.3%), amoxicillin in six (27.3%), and one each of amoxicillin/clavulanic acid (Clavamox, Zoetis, Parsippany-Troy Hills, NJ) and neomycin (4.5%). The median duration of lactulose administration at the time of fecal collection was 50.5 days (range, 1–842). The median duration of antibiotic administration was 45 days (range, 22–842).

Following initiation of medical management, the following clinical signs remained present: four (14.8%) with hyporexia or anorexia, three (11.1%) with neurologic signs (none with seizures), three (11.1%) with distinct GI signs, and three (11.1%) with lower urinary tract signs. Only two dogs developed new clinical signs between the onset of medical management and fecal collection–one with hyporexia/anorexia and one with lower urinary tract signs. Statistical analysis was performed to determine if there was a difference in bacterial abundances or in the DI between dogs who responded to medical management vs. those that did not—no statistical significance was identified.

In total, 22 (81.5%) dogs underwent surgical intervention for attenuation of CPSS including 14 (63.6%) with ameroid constrictor placement and eight (36.4%) with percutaneous transvenous coil embolization. Follow-up data was available in 20 (90.9%) dogs undergoing surgical intervention for the CPSS, and 13 (65.0%) had bloodwork findings suggestive of closure of the PSS based on interpretation of serum bile acids and hepatic synthetic factors.

Information pertaining to blood analyses performed during the same hospitalization for fecal collection can be found in Table 1. The bacterial abundance as measured in these dogs is available in Table 2. In total, 17 (63.0%) dogs in the study had a DI of > 2, which has previously been reported as the cutoff for dysbiosis in dogs (4). The overall median DI in this population of dogs was 3.02 (range 4.23–8.42). The median DI in dogs receiving antibiotics was 4.3 (range −4.23–8.42), and the median DI in dogs not receiving antibiotics was 1.52 (range −1.62–5.43) (*p* = 0.58).

**Table 1:** Blood analytes: Summarized data for clinicopathologic variables for the study population.

**Blood analyte**	**Number of cases**	**Median**	**Number of cases**	**Median**	**Reference**
	**tested at presentation**	**(range)**	**tested postoperatively**	**(range)**	**range**
**Serum biochemistry**					
Albumin (g/dL)	26	2.5 (1.8–3.6)	19	3.0 (2.4–4.1)	3.0–4.3
Globulins (g/dL)	26	3.45 (1.5–4.8)	N/A	N/A	2.6–4.7
Cholesterol (mg/dL)	25	178 (64–420)	14	229.5 (174–290)	74–255
BUN (mg/dL)	27	5 (2–13)	19	8.5 (4–26)	18–40
Glucose (mg/dL)	27	107 (51–121)	19	88 (80–195)	87–179
ALT (IU/L)	26	82.5 (31–516)	N/A	N/A	29–109
ALP (IU/L)	26	111 (41–441)	N/A	N/A	12–79
GGT (IU/L)	26	3 (2–13)	N/A	N/A	0–5
Total bilirubin (mg/dL)	26	0.4 (0.1–0.7)	N/A	N/A	0.1–0.7
**Complete blood count**					
PCV (%)	27	36.1 (28.0–50.5)	N/A	N/A	34–48
Total protein (g/dL)	26	5.95 (3.3–7.3)	N/A	N/A	6.6–8.4
White blood cells (x10E3/uL)	26	13.6 (9.5–20.0)	N/A	N/A	4.7–15.3
Neutrophils (x10E3/uL)	26	9.2 (3.8–17.8)	N/A	N/A	2.00–9.20
Lymphocytes (x10E3/uL)	26	9.5 (1.19–15.8)	N/A	N/A	1.05–8.00
Platelets (x10E3/uL)	26	196.5 (103–395)	N/A	N/A	169–480
**Liver function tests**					
Pre-prandial bile acids (μg/dL)	22	182.2 (0–318.8)	18	N/A	0–30
Post-prandial bile acids (μg/dL)	23	209.1 (69–382.3)	18	N/A	0–10
Resting ammonia (μmol/L)	13	140 (66–864.4)	N/A	N/A	<70 μmol/L^42^

*Post-operatively, only synthetic hepatic factors and serum bile acids were analyzed. Numerical values for post-operative serum bile acids were not recorded, but rather if there was an improvement or not as compared to the preoperative values*.

*BUN, blood urea nitrogen; ALT, alanine aminotransferase; ALP, alkaline phosphatase; GGT, gamma-glutamyl transferase; PCV, packed cell volume*.

**Table 2:** Bacterial abundances: Summary of the various bacterial abundances present in the study population.

**Bacteria**	**Abundance reference**	**Median**	**Number of dogs**	**Number of dogs within**
	**interval (log DNA/gram feces)**	**(range)**	**outside reference interval (%)**	**reference interval (%)**
Universal Bacteria	10.6–11.4	10.9 (10.43–13.22)	6 (22.2%)	21 (77.8%)
*Faecalibacterium*	3.4–8.0	7.28 (3.45–7.32)	2 (7.4%)	25 (92.6%)
*Turicibacter*	4.6–8.1	7.46 (3.25–9.13)	7 (25.9%)	20 (74.1%)
*Streptococcus*	1.9–8.0	6.05 (2.14–8.2)	1 (3.7%)	26 (96.3%)
*E. coli*	0.9–8.0	8.15 (0.88–8.77)	6 (22.2%)	21 (77.8%)
*Blautia*	9.5–11.0	11.23 (6.04–12.25)	14 (51.9%)	13 (48.1%)
*Fusobacterium*	7.0–10.3	9.69 (6.51–11.63)	8 (29.6%)	19 (70.4%)
*C. hiranonis*	5.1–7.1	3.07 (0.01–6.84)	19 (70.4%)	8 (29.6%)

*Reference intervals were established in 120 healthy dogs (4)*.

No significant effect on the DI from any of the components of medical management was noted. However, the abundance of E. coli was significantly positively affected by the use of metronidazole (*p* = 0.024). No significant effects on the DI or any individual bacterial species abundances were identified with amoxicillin, amoxicillin/clavulanic acid, or neomycin. Preoperative serum albumin had a significant negative impact on the DI (*p* = 0.009), whereas no other clinicopathologic variables had a significant relationship. C. hiranonis abundances were significantly positively affected by preoperative serum albumin (*p* = 0.035), serum GGT (*p* = 0.04), and serum cholesterol (*p* = 0.023). E. coli abundances were significantly negatively affected by increasing preoperative platelet count (*p* = 0.04), lymphocytes (*p* = 0.049), and BUN (*p* = 0.016).

## Discussion

The current pilot study is the first in the veterinary literature to describe evaluation of the dysbiosis index in a cohort of dogs diagnosed with CPSS. Given the fact that many dogs with CPSS are administered medications to mitigate the clinical signs secondary to CPSS and that these medications are known to affect overall dysbiosis, evaluating these interactions is important to further understand treatment effects on these patients (4, 15, 18–20). The overall rate of dysbiosis (DI >2) in the study population was relatively high at 63.0%, but no significant associations were identified between the DI and any individual component of medical management or clinical signs. Additionally, there was no significant association between the DI and clinical outcome or any of the clinicopathologic findings apart from a negative association with preoperative albumin. Additionally, significant associations between certain parameters and the abundance of individual bacterial species were also identified. This data allows us to accept a portion of hypothesis in this pilot study, that although a significant portion of these dogs were characterized with dysbiosis it did not affect clinical outcome. However, medical management was not significantly associated with the presence of dysbiosis.

The role of the human GI microbiome in chronic hepatopathies and HE is well established (10, 21). Significant differences have been shown in the colonic microbiota of human patients with cirrhosis or HE and healthy control individuals, and some authors suggest that the alterations in the microbiome in these patients may contribute to more significant clinical signs of HE or complications leading to mortality (12, 21). Investigation of the GI microbiome in dogs with hepatic pathology is more limited. A pilot study in 2015 conducted with 10 dogs revealed that one dog with chronic active hepatitis had Fusobacteria as the dominant bacterial phylum in the distal gut, as opposed to healthy dogs or dogs with distinct systemic pathology not involving the liver, in which the dominant organisms were Firmicutes or Proteobacteria, though clinical significance of this finding could not be determined (23). Given the sparsity of literature, the role of dysbiosis in relation to hepatic dysfunction or pathology, including CPSS, in dogs has yet to be elucidated.

All dogs in the present study had been medically managed prior to fecal sample collection with medications to reduce ammonia production and absorption in the gut and the manifestation of correlated clinical signs. All dogs were fed a therapeutic hepatic diet at the time of fecal collection. Moderately protein-restricted diets that provide high-quality protein (bioavailable and with a balanced amino acid composition) are recommended for dogs with CPSS, as these diets have been shown to reduce the severity of HE scores in dogs with CPSS (15, 16, 24). Though several studies on the effects of diet on intestinal microbiota in dogs have been published, none have been performed specifically with a reduced protein diet fed to dogs with hepatic pathology (24–27). These studies show that diet has the potential to cause statistically significant shifts in the fecal microbiota with variable size effect, corroborating the results of several human studies investigating the effects of diet on the microbiome (28–30). Given the other treatments administered in the dogs of this study, it is difficult to determine how significant the effects of the administered therapeutic hepatic diets were on the fecal microbiota of these dogs.

Lactulose was also administered to all dogs in the present study prior to fecal collection. Lactulose has been shown to cause a significant, reversible alteration of the microbiome in healthy dogs, but its effects on the microbiome have not been studied in dogs with any disease state (15). Additionally, the effects of lactulose administered concurrently with antibiotics has not been studied in dogs. Contrary to the aforementioned study in dogs, one study in humans showed that lactulose did not have a significant impact on the microbiome in patients with cirrhosis (31). Because all dogs in the present study were being treated with lactulose at the time of fecal sample collection, we cannot draw conclusions about the specific effect of lactulose in this population, though it may have contributed to the relatively high prevalence of dysbiosis (63.0%) in light of the previous canine study. This is an area that would benefit from potential future study.

No significant differences in the DI were noted between the 22 dogs (81.5%) receiving antibiotics and the five (18.5%) that were not. Previous studies performed in humans and dogs have shown both transient and long-lasting dysbiosis associated with antibiotic use, including the highly prevalent problem of Clostridium difficile in people (18, 32–34). Despite the lack of association with the DI, the present study identified a significant association between metronidazole and a greater abundance of E. coli (*p* = 0.024), in agreement with the results of a previous study investigating the use of metronidazole in healthy dogs (18). No significant correlation between the use of other antibiotics was identified in this study, though sample sizes were limited. The relationship between amoxicillin/clavulanic acid and the DI has previously been investigated in dogs, and no significant affect was shown (35).

Clostridium hiranonis has garnered attention in its role in intestinal health, as well as the conversion of bile acids in the gut. This bacterial species has a strong negative correlation with an increase in the dysbiosis index in dogs (4). In our study, C. hiranonis was below the canine reference range in 19 (70.4%) dogs, with a median value of 0.99. Our study population also exhibited significantly elevated pre- (median: 112.9, range 10.5–318.8) and post-prandial (median: 205.6, range 69–382.3) serum bile acid concentrations, consistent with what is typical for dogs with CPSS. The shunting of blood bypassing the normal hepatic recycling of bile acids in these dogs with CPSS is likely the main driving force behind this elevation in bile acids, but the prevalence of low abundances of C. hiranonis may play a role as well.

The abundance of C. hiranonis was also significantly positively correlated with preoperative serum albumin and cholesterol. While these findings may be incidental, they may also be associated with subclinical malabsorptive GI disease. It is the authors' observation that many dogs with CPSS are suspected to have concurrent GI disease at the time of presentation, in addition to reference of this concurrent process in a group of dogs with intrahepatic CPSS (36). Several studies have elucidated the correlation between inflammatory enteropathies and dysbiosis in dogs, and the above findings may support this association (5–7, 37). Further study is required to determine the individual role of fecal C. hiranonis in dogs with CPSS prior to medical management.

We identified a significant negative correlation between the abundance of E. coli and both pre-operative lymphocyte and platelet counts. Several studies in human medicine have investigated a link between the GI microbiome, macronutrient environment, and various immune cells, including B lymphocytes present in the gut-associated lymphoid tissue (GALT) (38, 39). Platelets, in addition to their role in primary hemostasis, have also been shown to be capable of immune defense against E. coli, while conversely the lipopolysaccharide associated with E. coli has been shown to be lethal to platelets (40, 41). Though these relationships have only been studied in humans, the negative correlation identified in our study between E. coli abundance and platelet counts may be the result of these effects. Further study focused on E. coli abundance and platelet and lymphocyte counts would be required to confirm this correlation in dogs.

In our study, no significant correlation was identified between the DI and the manifestation of clinical signs, either before or after medical management was initiated. This may be the case for several reasons; the clinical signs in dogs with CPSS may be more attributable to the shunting of blood bypassing the liver, increased production and absorption of ammonia, or alterations to the GI blood supply as opposed to intestinal dysbiosis. Additionally, our characterization of the microbiome was limited to fecal samples. Fecal samples have been shown to be significantly different from small intestinal samples, and the bacterial populations exhibit significant differences even within individual dogs in transit from the stomach to the colon (42). It may be the case that changes in the small intestinal microbiome are more clinically significant than those in the colon or the feces for dogs with CPSS. Finally, there are many protective mechanisms in place to prevent dysbiosis from cultivating clinical signs. These mechanisms include the intimate association between the intestines and the immune system, as well as the normal intestinal mucus layer that is present in healthy dogs and likely dogs with CPSS (as opposed to dogs suffering from chronic inflammatory enteropathies) (38, 39). If these protective functions were intact in the dogs in our study population, this may have also contributed to the lack of significant association with the DI and the clinical signs listed.

Interpretation of the microbiome in our study was performed with 16s rRNA qPCR, as determined by previous studies investigating the DI in dogs (4). This methodology is intended to look at the “functional core” of the microbiome in dogs, which is well preserved across differing individual dogs, dog breeds, and even between dogs and humans (3). Hence, changes in other bacterial taxa or species not included in this “functional core” may not have been identified in this population. Despite this potential limitation, we elected to utilize the qPCR as a way of avoiding overinterpretation of changes in the microbiome that may not be clinically relevant.

Our study had several limitations. The sample size of 27 dogs is relatively small, increasing the likelihood for type II error for our statistical analyses, though it is the only study to-date to specifically evaluate the fecal microbiota in dogs with CPSS. Though fecal sample collection was performed prospectively, data collection on outcome was performed retrospectively, and interpretation of clinical signs was often based on owner observations or referring veterinarian medical records. Additionally, the majority of dogs in our study were diagnosed with CPSS and started on medical management prior to referral to our institution, which precluded fecal sample collection before the initiation of medical management. Because medical management administration was performed at home, owner compliance was likely variable, and medical management strategies were diverse to start with as many referring veterinarians elected different protocols when initiating treatment for suspected CPSS. Unfortunately, there may be ethical implications to denying patients diagnosed with CPSS medical management to improve or mitigate clinical signs and side effects related to their diagnosis for study purposes.

In summary, this pilot is the first to describe the GI microbiome in dogs with CPSS. We identified that dysbiosis is common in dogs being medically managed for CPSS, despite some variability in the protocol being used. We did not observe a relationship between fecal dysbiosis and clinical signs or outcome in dogs diagnosed with CPSS. Areas of future study should include characterization of the microbiome and DI in dogs prior to the initiation of medical management, and isolating individual components of the medical management protocol with control groups to determine their individual contribution to the changes described here. These studies will need to be performed in such a way to avoid negative effects to patients which may require certain medications to maximize the likelihood of an uncomplicated clinical course following CPSS diagnosis. The DI and microbiome should also be described in dogs after having undergone surgical attenuation of their CPSS and following cessation of medical management. Though the DI did not appear to have any obvious effect on clinical outcomes in our study, controlled, prospective studies are warranted to further investigate this relationship as this may hold relevance for the treatment plan for dogs with CPSS.

## Data Availability Statement

The original contributions presented in the study are included in the article/supplementary material, further inquiries can be directed to the corresponding author/s.

## Ethics Statement

The animal study was reviewed and approved by University of Tennessee Institutional Animal Care and Use Committee (IACUC). Written informed consent was obtained from the owners for the participation of their animals in this study.

## Author Contributions

NS, CL, and XS drafted the manuscript. CL, KT, JL, and JS were all involved in study design and data collection. All authors were involved in the revision process. All authors contributed to the article and approved the submitted version.

## Conflict of Interest

The authors declare that the research was conducted in the absence of any commercial or financial relationships that could be construed as a potential conflict of interest.

## Publisher's Note

All claims expressed in this article are solely those of the authors and do not necessarily represent those of their affiliated organizations, or those of the publisher, the editors and the reviewers. Any product that may be evaluated in this article, or claim that may be made by its manufacturer, is not guaranteed or endorsed by the publisher.
